# Metabolic Engineering and Process Intensification for Muconic Acid Production Using *Saccharomyces cerevisiae*

**DOI:** 10.3390/ijms251910245

**Published:** 2024-09-24

**Authors:** Sinah Tönjes, Evelien Uitterhaegen, Ilse Palmans, Birthe Ibach, Karel De Winter, Patrick Van Dijck, Wim Soetaert, Paul Vandecruys

**Affiliations:** 1 Centre for Industrial Biotechnology and Biocatalysis (InBio.be), Department of Biotechnology, Faculty of Bioscience Engineering, Ghent University, 9000 Ghent, Belgium; sinah.toenjes@ugent.be (S.T.);; 2 Bio Base Europe Pilot Plant (BBEPP), 9042 Ghent, Belgium; 3 Laboratory of Molecular Cell Biology, Institute of Botany and Microbiology, KU Leuven, 3001 Leuven, Belgium; ilse.palmans@kuleuven.be (I.P.); patrick.vandijck@kuleuven.be (P.V.D.); paul.vandecruys@kuleuven.be (P.V.)

**Keywords:** muconic acid, *Saccharomyces cerevisiae*, yeast cell factory, metabolic engineering, *in situ* product recovery, reactive extraction

## Abstract

The efficient production of biobased organic acids is crucial to move to a more sustainable and eco-friendly economy, where muconic acid is gaining interest as a versatile platform chemical to produce industrial building blocks, including adipic acid and terephthalic acid. In this study, a *Saccharomyces cerevisiae* platform strain able to convert glucose and xylose into *cis*,*cis*-muconic acid was further engineered to eliminate C2 dependency, improve muconic acid tolerance, enhance production and growth performance, and substantially reduce the side production of the intermediate protocatechuic acid. This was achieved by reintroducing the *PDC5* gene and overexpression of *QDR3* genes. The improved strain was integrated in low-pH fed-batch fermentations at bioreactor scale with integrated *in situ* product recovery. By adding a biocompatible organic phase consisting of CYTOP 503 and canola oil to the process, a continuous extraction of muconic acid was achieved, resulting in significant alleviation of product inhibition. Through this, the muconic acid titer and peak productivity were improved by 300% and 185%, respectively, reaching 9.3 g/L and 0.100 g/L/h in the *in situ* product recovery process as compared to 3.1 g/L and 0.054 g/L/h in the control process without ISPR.

## 1. Introduction

To transition the current fossil fuel-based economy to a more resilient biobased economy focused on sustainability, alternative production processes for everyday chemicals are required. Microbial production of platform molecules used to synthesize these chemicals offers a valuable alternative in this respect. The biobased production of 2,4-hexadienedioic acid, also referred to as muconic acid (MA), has attained attention over the last decade. This unsaturated dicarboxylic acid with two reactive dicarboxylic groups can be used to produce a diverse set of polymers, for example, polyesters [1]. In addition, MA can be converted into other platform chemicals of great significance. For example, MA can be hydrogenated to yield adipic acid, one of the monomers in nylon-6,6 [2]. Moreover, MA can be converted into levulinic acid, another platform chemical with a projected market size of 2400 tons yearly by 2025 [3].

Biobased synthesis of MA, more specifically the *cis*,*cis*-MA isomer (ccMA), has been successfully achieved through the metabolic engineering of various microorganisms. The synthesis is either based on the bioconversion of aromatic (lignin-derived) feedstocks or on *de novo* synthesis from carbohydrates. The former has resulted in yields of up to 85 g/L [4], where the main challenge remains the complexity of lignin as a feedstock, which requires extensive upstream purification [5,6]. The highest reported titer from *de novo* production to date is 88.2 g/L, accomplished with *Corynebacterium glutamicum* using glucose as the carbon source and maintaining the pH at 7.0 [7]. Yeast microbial cell factories are an appealing alternative due to, amongst other things, their high tolerance towards organic acids at low pH, resistance towards toxic inhibitors, and the possibility of them to utilize carbon sources from second-generation biomass [8,9,10]. Although research on non-conventional yeasts has gained momentum [11], most research to establish a eukaryotic production host has focused on *Saccharomyces cerevisiae*. However, the production of ccMA in *S. cerevisiae* is constrained by the significant inhibitory effect of MA on yeast viability, resulting in maximum titers, yields, and productivities in a fed-batch 2 L bioreactor of 22.5 g/L, 77 mg/g glucose, and 0.191 g/L/h, respectively [12]. While these results were achieved in buffered conditions at pH 6.0, the toxicity at low pH is significantly increased, as ccMA exists primarily in its protonated form, allowing it to diffuse into the cells. In unbuffered conditions, concentrations as low as 5 g/L ccMA caused a significant reduction of 43% in the maximum specific growth rate of the laboratory reference strain *S. cerevisiae* CEN.PK 113-7D [13]. Despite this, low-pH fermentation remains attractive, as medium neutralization requires high amounts of acids and bases, typically resulting in significant salt waste, and can therefore compromise economic feasibility [14,15].

In that respect, continuous extraction of organic acids during fermentation in *in situ* product recovery (ISPR) processes has emerged as an attractive technique to reduce product inhibition and increase fermentation performance. Here, reactive extraction using organic phases containing amines, phosphorous compounds, and ionic liquids has been described to efficiently separate organic acids from fermentation broth. This, moreover, leads to a partial purification and concentration of the target product in the organic phase and can hence reduce downstream purification efforts [16]. This approach has been previously evaluated for MA production using *S. cerevisiae*, where adding polypropylene 400 to shake flask fermentations extracted MA but did not further improve strain performance [17]. In another study, a biocompatible reactive extraction mixture was developed containing CYTOP 503 in canola oil and was successfully applied to fermentation processes at the shake flask level with *S. cerevisiae*, resulting in a growth and MA titer improvement of 44% and 18%, respectively. A proof of the strategy at 10 L bioreactor scale led to a maximum MA titer of 4.33 g/L at pH 4.0 [18].

**Figure 1:**
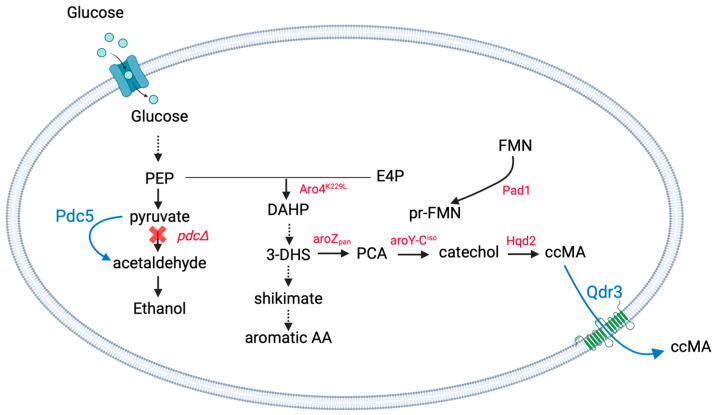
Schematic representation of the genetic targets engineered to establish muconic acid (MA) production in *S. cerevisiae* strain TN22 (red), and the modifications performed in this study (blue) to enhance its performance. In TN22, MA production was established by the expression of a 3-dehydroshikimate (DHS) dehydratase from *P. anserina* (aroZ_pan_), protocatechuic acid (PCA) decarboxylase (PCAD) from *K. pneumoniae* (aroY-C^iso^), and oxygen-consuming catechol 1,2-dioxygenase (CDO) from *C. albicans* (Hqd2). Feedback inhibition by aromatic amino acids for entry of carbon into the shikimate pathway was alleviated by expressing a feedback-resistant DAHP-synthase (Aro4^K229L^) and the production of prenylated flavine mononucleotide (prFMN), a co-factor of PCAD, was enhanced by overexpressing *PAD1*. Moreover, ethanol production was eliminated by the deletion of *PDC1*, *PDC5* and *PDC6*. In this work, *PDC5* was reintegrated to eliminate the C2 dependency for ccMA production and the multidrug transporter encoded by *QDR3* was expressed to enhance MA export (Created with BioRender.com).

The present study uses a previously engineered MA-producing *S. cerevisiae* strain, originally constructed by Nicolaï and coworkers [17], as a basis for further engineering. The modifications present in that original strain are shown in Figure 1. This strain can convert glucose and xylose to ccMA as it holds a heterologous pathway for MA biosynthesis from 3-dehydroshikimate (DHS). DHS dehydratase from *Podospora anserina*, protocatechuic acid (PCA) decarboxylase (PCAD) from *Klebsiella pneumoniae*, and oxygen-consuming catechol 1,2-dioxygenase (CDO) from *Candida albicans* were expressed, while ethanol production was eliminated by deletion of *PDC1*, *PDC5*, and *PDC6*. In addition, feedback inhibition for the entry into the shikimate pathway was eliminated by the integration of a feedback-resistant DAHP synthase. Moreover, the overexpression of *PAD1* in this strain enhanced the production of prenylated flavine mononucleotide (prFMN), a cofactor of PCAD. To alleviate the growth on glucose of this pyruvate-decarboxylase negative (Pdc^−^) strain, an internal deletion of *MTH1*, *MTH1*^ΔM41-T78^_,_ was introduced [19,20,21]. This strain, referred to as *S. cerevisiae* TN22, served as the starting point for the current work, where engineering efforts were focused on the reduction of the C2 dependency and the improvement of ccMA tolerance (Figure 1). The improved strain was evaluated in a fed-batch fermentation at bioreactor scale with ISPR, aiming to further reduce product inhibition and enhance the process performance at low-pH to contribute to a more sustainable production of MA and, by extension, other biobased platform chemicals.

## 2. Results

### 2.1. Elimination of C2 Dependency for ccMA Production by Reintroduction of PDC5

**Figure 2:**
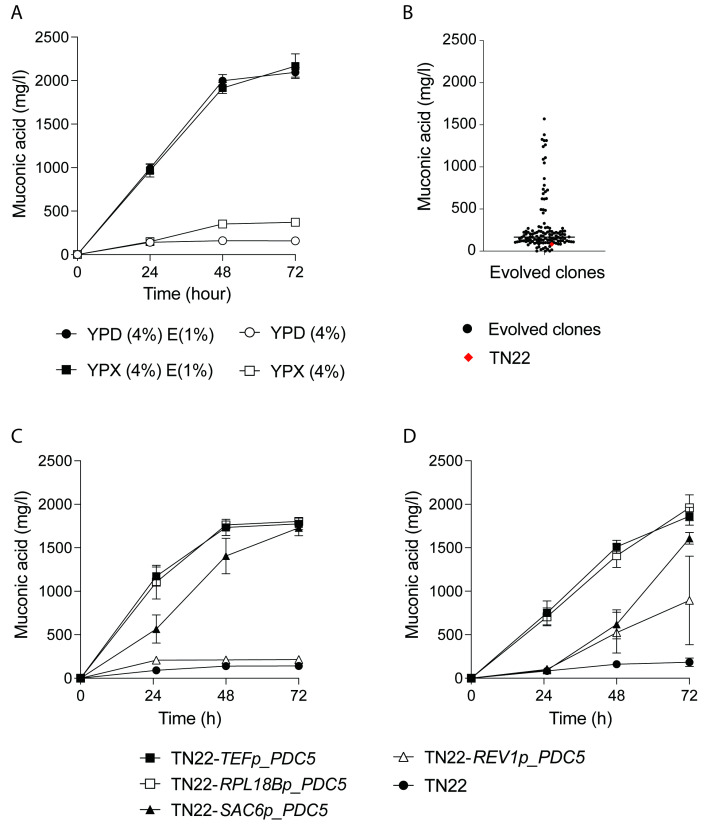
Relieving the C2 dependency of *S. cerevisiae* TN22. (**A**) ccMA production at shake flask level by *S. cerevisiae* TN22 in yeast extract-peptone 4% dextrose (YP4%D) supplemented with 1% ethanol (●) and yeast extract-peptone-xylose (YP4%X) supplemented with 1% ethanol (■), YP4%D (◯), and YP4%X (◻). (**B**) ccMA production by 129 evolved clones (black), compared to TN22 (red) after 72 hours in YP4%D. (**C**,**D**) Effect of *PDC5* overexpression on MA production in *S. cerevisiae* TN22. Strains were fermented at shake flask level in a glucose-containing medium, YP4%D (**C**), or xylose-containing medium, YP4%X (**D**). TN22 (●) was compared to strains overexpressing *PDC5*, in order of increasing promoter strengths: TN22 *REV1p_PDC5* (△), TN22 *SAC6p_PDC5* (▲), TN22 *RPL18Bp_PDC5* (◻), TN22 *TEF1p_PDC5* (■). Results are the means of three biological replicates. Error bars show the standard deviation at each time point.

Fermentation trials with the previously constructed MA-producing host strain, *S. cerevisiae* TN22, demonstrated its dependence on an endogenously added C2 carbon source, e.g., ethanol, to support ccMA production from glucose or xylose (Figure 2A). When supplemented with 1% ethanol, fermentation in yeast extract-peptone-dextrose (YP4%D) and yeast extract-peptone-xylose (YP4%X) yielded titers of 2.09 g/L and 2.17 g/L ccMA, respectively. In contrast, only 0.158 and 0.372 g/L ccMA were produced in YP4%D and YP4%X without ethanol. In addition, growth in media without ethanol was limited. To address its C2 dependence, *S. cerevisiae* TN22 was subjected to adaptive laboratory evolution (ALE). Ten populations were cultured in media where ethanol concentrations were gradually lowered. For five of these populations, prior to evolution, additional genetic diversity was introduced by performing EMS mutagenesis. Over a period of three months, a total of 15 media transfers were performed, alternating glucose and xylose as carbon sources to avoid undesired trade-offs. At regular intervals, cells were plated. A total of 129 evolved clones were tested for their ability to produce ccMA in shake flasks in YP4%D, without the addition of ethanol (Figure 2B). Upon fermentation completion, 19 evolved clones yielded a ccMA titer higher than 0.5 g/L. The top-performing clone produced 1.57 g/L ccMA from 4% glucose, corresponding to a yield of 39.9 mg/g glucose and approaching the yield of the unevolved strains on YPD supplemented with ethanol (41.8 mg/g glucose and ethanol). However, for the top-performing mutants, xylose conversion into ccMA in YPD was compromised, delivering titers below 0.350 g/L ccMA when YP4%X was fermented.

Instead, as an alternative approach to address the ethanol dependency of *S. cerevisiae* TN22, *PDC5* was reintroduced. Four constitutive promoters of variable strengths were used. Fermentation profiles are shown in Figure 2C,D. *PDC5* expression under the *TEF1* and *RPL18B* promoters showed restoration of ccMA production in YP4%D without the addition of ethanol. During the first phase of the fermentation, ethanol was produced (24 h), which was subsequently completely consumed to produce ccMA. Moreover, growth of the modified strains was significantly improved compared to *S. cerevisiae* TN22. Strain *S. cerevisiae* TN22 *RPL18Bp_PDC5* was chosen as a starting strain for further engineering efforts.

### 2.2. QDR3 Overexpression Improves ccMA Production and Limits Byproduct Formation

Excessive accumulation of pathway intermediates is undesirable for a microbial cell factory as it can directly lower product yields. In addition, these intermediates can also complicate extraction procedures as they often chemically resemble the target chemical, thereby complicating purification efforts. While catechol remained below detection limits for *S. cerevisiae* TN22, it still produced 229.8 mg/L of the pathway intermediate PCA. This can have a direct negative effect on the product yield. Another limitation of this production host is the limited tolerance of *S. cerevisiae* towards MA [10].

**Figure 3:**
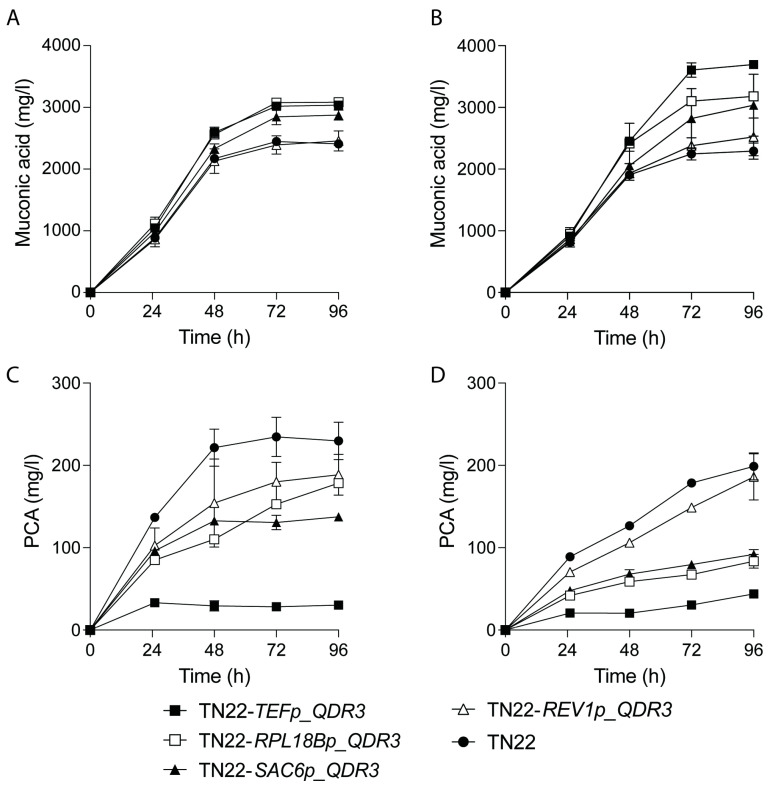
Overexpression of *QDR3* in *S. cerevisiae* TN22 improves ccMA (**A**,**B**) and PCA (**C**,**D**) production. Strains were fermented in glucose-containing medium, YP4%D1%E (**A**,**C**), or xylose-containing medium, YP4%X1%E (**B**,**D**). *S. cerevisiae* TN22 (●) was compared to strains overexpressing *QDR3*, in order of increasing promoter strengths TN22 *REV1*p_*QDR3* (△), TN22 *SAC6*p_*QDR3* (▲), TN22 *RPL18B*p_*QDR3* (◻), TN22 *TEF1*p_*QDR3* (■). Results are the means of three biological replicates. Error bars show the standard deviation at each time point.

One strategy to mitigate the toxicity of a compound involves its extracellular export. We hypothesized that lowering the intracellular ccMA concentrations by improving its export could substantially increase ccMA tolerance and reduce the build-up of PCA. To this end, we overexpressed *QDR3*, a gene encoding a multidrug transporter known to confer tolerance to MA [22]. To export ccMA, *QDR3* was overexpressed under four constitutive promoters of variable strengths. Figure 3A,B compare ccMA production and PCA accumulation of engineered strains to *S. cerevisiae* TN22 during the fermentation of glucose- and xylose-based media supplemented with ethanol. ccMA titers increased with the strength of the promoters, with *S. cerevisiae* TN22 *TEF1*p_*QDR3* yielding 3.04 g/L and 3.70 g/L ccMA on glucose- and xylose-containing media, respectively. In addition, this modification lowered PCA concentrations, with 30.3 mg/L and 43.8 mg/L in glucose- and xylose-containing media, respectively, compared to 229.8 mg/L and 198.8 mg/L for the unmodified *S. cerevisiae* TN22 (Figure 3C,D).

### 2.3. Combined Overexpression of PDC5 and QDR3 Enables Improved ccMA Production without Ethanol Supplementation

**Figure 4:**
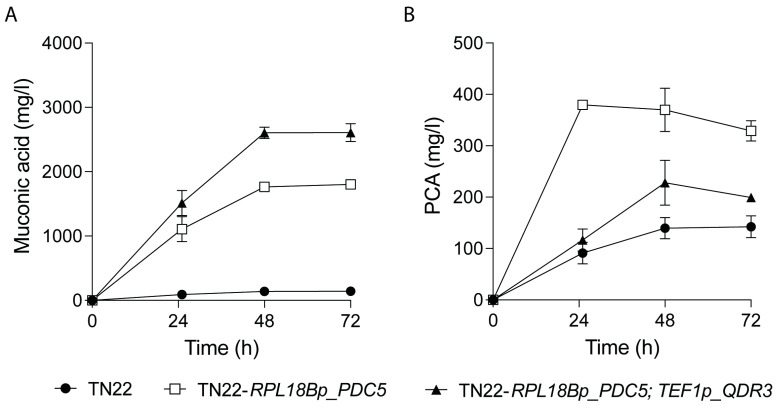
Overexpression of *QDR3* and *PDC5* in *S. cerevisiae* TN22 results in improved ccMA (**A**) and PCA (**B**) production. Strains were fermented in YP4%D. *S. cerevisiae* TN22 (●) was compared to *S. cerevisiae* TN22 *RPL18Bp*_*PDC5* (◻) and *S. cerevisiae* TN22 *RPL18Bp_PDC5*; *TEF1p*_*QDR3* (▲). Results are the means of three biological replicates. Error bars show the standard deviation at each time point.

Since both *PDC5* and *QDR3* overexpression were beneficial for ccMA production, both modifications were combined in one strain, yielding strain *S. cerevisiae* TN22 *RPL18Bp_PDC5*; *TEF1p_QDR3*. ccMA and PCA production of this strain during shake flask fermentations of YP4%D are shown in Figure 4, yielding a final ccMA titer of 2.61 g/L MA, with a yield of 65 mg/g glucose and a productivity, calculated over the first 48 hours, of 54 mg/L/h.

### 2.4. In Situ Product Recovery Significantly Improves Fermentation Performance in Bioreactors

**Figure 5:**
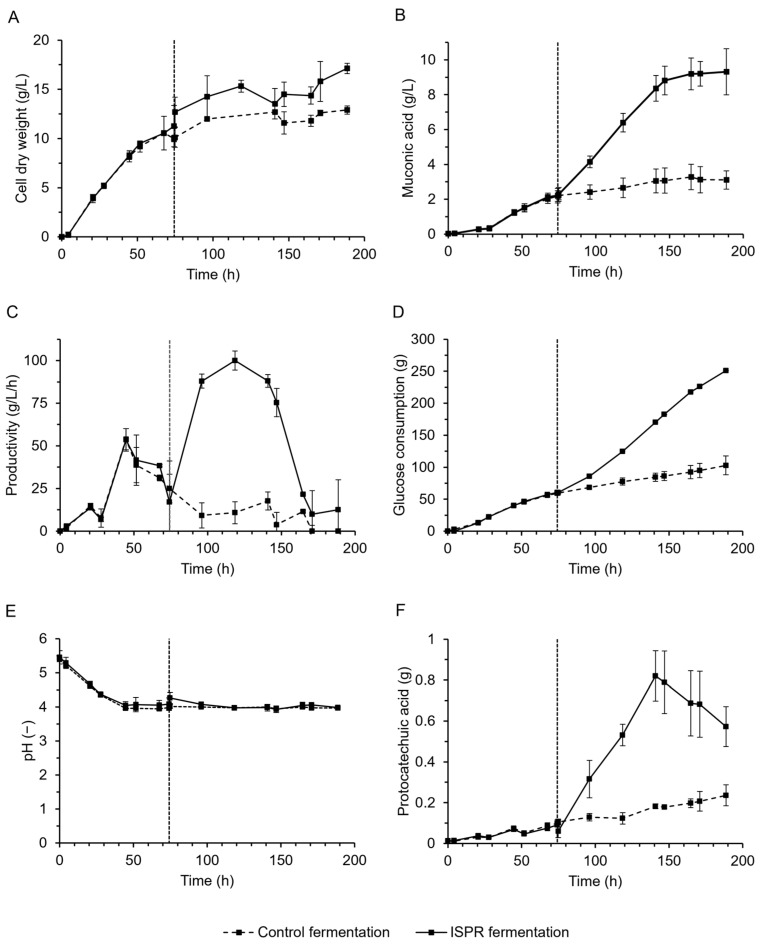
MA fermentations in 2L bioreactors using *S. cerevisiae* TN22 *RPL18Bp_PDC5*; *TEF1p_QDR3*. Solid lines represent *in situ* product recovery (ISPR) fermentations, where 20% of solvent (*v*/*v*) composed of CYTOP 503 (12.5 v%) in canola oil was added after 74 h of fermentation (vertical line). Dashed lines represent control fermentations. Cell dry weight (CDW) (**A**), MA titer (**B**), productivity (**C**), glucose consumption (**D**), pH (**E**), and PCA titer (**F**) are displayed as mean values of biological duplicates. Error bars show the deviations at each time point.

The improved strain was evaluated in fed-batch fermentations with continuous control of process parameters. Four parallel fermentations were executed in 2 L bioreactors, with two reactors employing ISPR, allowing a direct assessment of the influence of the extraction compared to the control reactors. Initially, all four reactors were operated under the same conditions. The starting pH was 5.5 and slowly decreased due to the production of MA, reaching 4.0 after approximately 45 h, where it was kept at by adding NaOH to the fermentation. After 74 h of fermentation, in all four reactors, a stagnation of growth at a cell dry weight (CDW, Figure 5A) of 10.6 ± 1.4 g/L, and of MA production at a titer of 2.2 ± 0.3 g/L (Figure 5B) was detected. This was also reflected in a sharp decrease in productivity (Figure 5C). At that point, in two of the reactors, a reactive extraction mixture was added to initiate ISPR. Thereupon, 39% of the MA was extracted into the organic phase. Consequently, growth, production, glucose consumption, and productivity readily increased compared to the control reactors (Figure 5).

The end-parameters of the fermentations are summarized in Table 1. The MA titer, productivity, and CDW were increased by 300%, 299% and 133%, respectively, reaching 9.3 ± 0.9 g/L, 49.4 ± 4.6 mg/L/h, and 17.1 ± 0.5 g/L in the ISPR reactors compared to 3.1 ± 0.7 g/L, 16.5 ± 3.8 mg/L/h, and 12.9 ± 0.4 g/L in the control reactors. In the ISPR reactors, glucose consumption was increased due to enhanced growth and production, resulting in the addition of more feed, hence an increased volume. Consequently, comparing the absolute amounts of MA, an improvement of 384% was reached in the ISPR reactors, producing 12.3 ± 1.3 g of MA compared to 3.2 ± 0.5 g in the control fermentations. After 119 h, the ISPR reactors reached a peak productivity of 0.1 ± 0.006 g/L/h, whereafter a sharp decrease was detected upon reaching inhibitory levels of MA again, also resembled by renewed stagnation of the MA titer (Figure 5B,C).

The production of the side-product PCA remained below 1 g/L in all reactors, despite the positive influence in the ISPR fermentations, which increased the production of PCA 2.3 times to a total of 0.57 ± 0.1 g compared to 0.24 ± 0.05 g in the controls (Figure 5E). The corresponding ratio of PCA-to-MA was as low as 0.08 in the control reactors and further decreased by 18% to 0.06 by implementing ISPR.

## 3. Discussion

In this work, the previously constructed MA-producing host strain *S. cerevisiae* TN22 [17] was further improved. To abolish ethanol formation as a byproduct, which diverts carbon away from MA, pyruvate decarboxylase-encoding genes were deleted in this strain. Therefore, like other Pdc^−^ strains, it is unable to grow in excess glucose, a defect likely caused by repression of the respiratory metabolism and a lack of cytosolic C2 supply [20]. Even though Nicolaï and coworkers engineered a mutation known to alleviate this defect, *MTH1*^ΔM41-T78^ [17], strain *S. cerevisiae* TN22 only showed limited growth with barely any ccMA production in medium without a C2 source (0.158 g/L in YP4%D). Since the requirement of ethanol addition constrains the industrial applicability of this MA production host, we attempted to address this defect by subjecting TN22 to ALE. Over the course of 15 rounds, several populations were transferred in media with ethanol concentrations that were gradually lowered. Fast-growing clones were selected for evaluation and yielded multiple promising clones, with the best isolate delivering a titer of 1.57 g/L ccMA from 4% glucose. Unfortunately, these promising isolates failed to substantially improve MA production in a xylose-containing medium without the addition of a C2 source. Two conclusions can be drawn from these observations. First, mutations that alleviate C2 dependency for MA production can be carbon source-dependent. Second, adaptive mutations that overcome the C2 auxotrophy of Pdc-negative strains for the utilization of xylose seem to be harder to acquire than mutations that allow the utilization of glucose. Since the ability to utilize xylose as a carbon source is vital for any industrial application of an MA-producing host strain, an alternative strategy was explored. The ability to produce ethanol was partially restored by the reintroduction of *PDC5*. A set of four promoters of variable strengths was used to express *PDC5*, and expression under an *RPL18B* promoter was sufficient to support MA production when glucose or xylose served as carbon sources. Since no further improvement in MA yield, productivity, or titer could be observed when the stronger *TEF1* promoter was applied, *S. cerevisiae* TN22 *RPL18Bp_PDC5* was chosen for further engineering efforts.

We subsequently increased tolerance to MA by enhancing its efflux. To this end, the multidrug transporter encoded by *QDR3*, known to mediate MA export, thereby conferring MA tolerance [22], was overexpressed. With increasing promoter strengths applied, MA titers of the engineered strains increased, with expression under a *TEF1* promoter resulting in the highest MA titers. Conversely, accumulation of the pathway intermediate PCA decreased with stronger promoter strengths. This reduction in byproduct formation is crucial for process economics, as it minimizes carbon diversion to unwanted products and simplifies product purification. Given the beneficial nature of this modification, it was engineered in strain *S. cerevisiae* TN22 *RPL18Bp*_*PDC5*, where similar improvements were observed. Given that the overexpression constructs with the highest promoter strengths yielded the largest improvements, at this moment it remains unclear whether further increasing *QDR3* expression, i.e., by integrating multiple copies of the overexpression constructs, may further improve the production host.

The improved *S. cerevisiae* TN22 *RPL18Bp_PDC5*; *TEF1p_QDR3* strain was subsequently implemented in fed-batch fermentations in 2 L bioreactors, reaching an end titer of 3.1 g/L with a peak productivity of 53.9 mg/L/h, which was in line with the shake flask experiments. However, product inhibition was distinct, and at a titer of 2.2 g/L of MA, production and growth stagnated and productivity had sharply decreased. At this timepoint, the pH of the medium had dropped to 4.0 from the initial value of 5.5. This observation of product inhibition of MA is in accordance with other studies (Table 2) showing that the highest performances were reached by neutral-pH fermentation, while low-pH fermentation has been limited by product inhibition. Toxicity towards *S. cerevisiae* was observed in fermentations without pH control at titers as low as 2.9 g/L [17] and 5 g/L [13]. Also, in other yeast species such as *P. occidentalis*, growth was completely stopped after a drop of pH to 2.0 and could only be restored partly at pH 3.5-4.0. Titers of MA in that case were reduced to 7.2 g/L compared to 38.8 g/L at a neutral pH [8]. The reason for the pronounced inhibition is directly linked to the dissociation behavior of MA being protonated at pH levels below pH 4.0 (pKa_1_: 2.9, pKa_2_:4.0) [23], enabling the diffusion into the cells.

To address the product inhibition, reactive extraction emerged as an attractive strategy to extract organic acids from low-pH fermentation broth, as commonly applied extractants in the literature show enhanced performance for protonated organic acids [36,37]. One constraint is reported to be the toxicity of the extractants to the microorganisms, but mixing extractants with biocompatible diluents can reduce toxicity and yield mixtures that can directly be added to the fermentation broth [38]. In our previous study, such a mixture containing the phosphorous based extractant CYTOP 503 (12.5 v%) and canola oil was developed and proved to be non-toxic for *S. cerevisiae* while retaining high extraction capacity for MA [18]. These findings are in line with the here attained data, and no negative interference with the new yeast strain was found. On the contrary, all performance parameters were significantly increased when ISPR was applied, including MA production, productivity, growth, and yield. To that end, an overall maximum titer of 9.3g/L was obtained in the ISPR fermentation.

Nevertheless, at the end of the fermentation, product inhibition was found recurrent, and while the results prove the effectiveness of ISPR, they also reveal the limitations of the ISPR process in a batch mode. In this respect, various options to industrially apply ISPR based on reactive extraction are described, where a continuous separation with a constant inflow of fresh organic phase and a constant outflow of loaded organic phase allows further process intensification. This can be achieved internally in the bioreactor with specialized equipment or externally by circulating the broth or fermentation supernatant over an external extraction unit [39,40,41]. Here, resource recycling is crucial, and future research should focus on efficient back-extraction techniques for the recovery of the organic acid and to allow the recycling of the reactive extraction mixture. In parallel, strain engineering efforts should be focused on increasing MA tolerance at low pH to enhance the overall fermentation performance to ultimately yield an economic process. ALE in *S. cerevisiae* presents an attractive approach to enhancing tolerance to ccMA, as it has been successfully applied to improve tolerance to other compounds such as acetic acid and lactic acid tolerance [42,43]. Notably, increases in the copy number of *QDR3* were previously observed during laboratory evolution experiments on adipic acid and glutaric acid, two other dicarboxylic acids [22].

In conclusion, in this study, an MA producing strain was successfully engineered with enhanced MA tolerance, production, and growth, while substantially reducing the side production of the intermediate PCA. Since organic acid production is limited by product inhibition at low-pH, ISPR was successfully applied to fermentation processes using the improved strain. By adding an organic reactive extraction mixture to the process, MA was continuously extracted, resulting in significant improvements in MA production and growth. This can serve as a basis for a sustainable, biobased production of MA and, by extension, other organic acids at low pH. Future research should investigate specialized industrial equipment for continuous operation, as well solvent recycling and reuse.

## 4. Materials and Methods

### 4.1. Microorganisms, Plasmids and Culture Media

Yeasts were cultivated in YPD medium (2% [*w*/*v*] d-glucose, 2% [*w*/*v*] bacteriological peptone (Oxoid/Thermo Fisher Scientific, Pittsburgh, PA, USA), 1% [*w*/*v*] yeast extract granulated (Merck/Kerry Ingredients & Flavours Ltd., Tralee, Ireland)) shaking at 200 rpm and at 30 °C. For solid nutrient plates, 1.5% (*w*/*v*) Difco agar bacteriological (Becton Dickinson and Company, Franklin Lakes, NJ, USA) was added. *Escherichia coli* cells (TOP10; Invitrogen) were grown at 37 °C in Lennox broth (LB) (Merck, Rahway, NJ, USA). For solid nutrient plates, 1.5% (*w*/*v*) Bacto agar was added. When required, one or a combination of the following antibiotics were added to solid agar plates: 20 mg/L nourseothricin, 100 mg/L geneticin, or 100 mg/L ampicillin. For long-term storage of yeast and bacterial strains, 30% glycerol or 25% glycerol were added to YP or LB medium, respectively, before storage at −80 °C. The plasmids and *Saccharomyces cerevisiae* strains used in this study are listed in Table 3 and Table 4.

### 4.2. Molecular Biology Methods

*E. coli* cells were rendered chemically competent and transformed by the method described by Green and Rogers [44]. Yeast cells were transformed by electroporation [45]. Genomic DNA was extracted by phenol/chloroform/isoamyl-alcohol extraction [46]. Diagnostic PCR was performed using OneTaq DNA polymerase (New England Biolabs, Ipswich, MA, USA), while Q5 high-fidelity DNA polymerase was used to amplify donor DNA fragments or genomic regions for sequencing and cloning purposes (New England Biolabs). The Nucleospin^®^ Plasmid Easy Pure kit (Macherey-Nagel, Düren, Germany) was used for plasmid purification, and PCR amplicons were purified, when required, using the Nucleospin Gel and PCR Clean-up kit (Macherey-Nagel). Sanger sequencing was performed by Eurofins genomics and the obtained sequencing reads were aligned using the CLC Main Workbench (Qiagen, Venlo, The Netherlands).

### 4.3. Overexpression of PDC5 and QDR3

To generate the overexpression cassettes, the open reading frames (ORFs) of *PDC5* and *QDR3* and the selected promoter/terminator regions were amplified from laboratory strain *S. cerevisiae* S288c (MATa/alpha) using primers listed in Table S1. NEBbuilder HiFi DNA Assembly (New England Biolabs) was used to assemble the PCR fragments into plasmid backbone pCR2.1 (Thermo Fisher Scientific). Upon cloning and verification by Sanger sequencing, overexpression constructs were amplified using primers holding 65 bp tails, homologous to the desired genomic integration site. Using a CRISPR/Cas9-based approach, *QDR3* was integrated at integration site IS4.2 (NC_001136.10:g.980427_980458), while *PDC5* was integrated at integration site IS12.2 (NC_001144.5:g.839677_839682). Cas9 was expressed from plasmid pTEF-Cas9-KanMX, and specific gRNAs were assembled in XhoI-EcoRV-digested plasmid pgRNA-uni-NAT, a CRISPR/CAS9-expression system that was previously employed successfully [47,48]. Integration of the cassettes at the desired location was checked by PCR using primers flanking the integration sites and the absence of mutations was verified by Sanger sequencing. Successful transformants were grown for at least 25 generations under non-restrictive conditions to lose the Cas9 expression and gRNA plasmids. Plasmid loss was confirmed by spot assay on YPD supplemented with geneticin or nourseothricin.

### 4.4. Fermentations

For shake flask fermentations, yeast cells were grown in YP medium supplemented with glycerol (2%) and ethanol (2%) for 3 days. Cells were washed once with YP medium, pitched at an OD600 of 4, and fermented in Erlenmeyer flasks in an orbital shaking incubator at 200 rpm at 30 °C. For fed-batch fermentation, 2 L glass bench-top bioreactors (Eppendorf DASGIP^®^ Parallel Bioreactor system) with an initial working volume of 1 L were used. Here, a seed train consisting of two steps was performed. For the first step, 500 mL baffled shake flasks containing 20% of YP2.5%D were inoculated with 1 mL of the yeast culture from a cryovial stored at −80 °C, incubated at 200 rpm (orbit 5.1 cm) and 30 °C to an OD600 ≥ 5. Subsequently, a second seed step in 1000 mL baffled shake flasks and otherwise similar conditions were inoculated with 5% of the seed culture (*v*/*v*) and cultivated for 25 h before inoculating the main fermenter with 5% of the second seed culture (*v*/*v*). Process parameters were continuously monitored and controlled, ensuring close follow-up and minimizing variability. The temperature was maintained at 30 °C and the dissolved oxygen (DO) was kept at 30% by automated control of the stirrer speed (150–1200 rpm) and the airflow (0.1 vvm). The starting pH was 5.5 and dropped naturally to 4.0 due to the production of MA, where it was kept constant through the addition of 1M NaOH. Upon depletion of the batch glucose (50 g/L), a continuous feeding of 600 g/L of glucose was applied at a feed rate of 2.0–3.5 mL/h. For ISPR trials, 20% (*v*/*v*) of a solvent mixture consisting of 12.5% CYTOP 503 in canola oil was added to the bioreactors upon reaching an inhibitory concentration of MA. At regular intervals, samples were withdrawn for pH measurements, CDW measurements, and metabolite determinations (see Section 4.5). Shake flask fermentations were performed in independent triplicates, and fermentation at bioreactor scale in independent duplicates. The results are reported as average values with standard deviations.

### 4.5. Analytics

Metabolites were determined using high-performance liquid chromatography (HPLC), where either an Agilent MetaCarb 87H column, maintained at 60 °C, or a MetaCarb 67H column (connected to a varia 5244GC precolumn), maintained at 40 °C, with 2.5 mM H_2_SO_4_ as mobile phase was used. Sugars and ethanol were detected by a refractive index detector (RID), while MA, PCA, and catechol were detected using a UV detector (SPD-20A, Shimadzu, Kyoto, Japan) at 270 nm. Alternatively, MA and PCA were analyzed using a Zorbax Eclipse Plus C18 column (4.6 × 100 mm, 3.5 Micron). The mobile phase in this case was 0.1% TFA in MilliQ water (A) mixed with 0.1% TFA in acetonitrile (B) with a flow of 0.75 mL/min and a column temperature of 40 °C. The following gradient was applied: 95% A, 5% B from 0–15 min, and 95% B, 5% A from 16–26 min. The compounds were measured with a DAD detector at a wavelength of 230 nm. Prior to the analysis, cells were pelleted by centrifugation and the supernatant was injected. Since ccMA isomerizes at low pH to the *cis*,*trans* isomer of MA [49], for low-pH fermentations, the sum of both isomers was reported.

CDW was determined gravimetrically by centrifuging 5 mL of the culture broth (5 min, 14,000 rpm), whereafter the resulting cell pellet was washed with physiological water, resuspended in distilled water, and transferred to a moisture analyzer (MA37, Sartorius) to dry the sample to a constant weight.

## Figures and Tables

**Table 1:** Performance parameter of fed-batch fermentations using *S. cerevisiae* TN22 *RPL18Bp_PDC5*; *TEF1p_QDR3* in 2 L bioreactors comparing processes with and without ISPR. In ISPR processes, an organic reactive extraction mixture containing 12.5 v% of CYTOP 503 in canola oil was added to the process. Mean values of biological duplicates are displayed with the corresponding standard deviation.

Parameter	Control Fermentation	ISPR Fermentation
Duration (h)	188.6	188.6
MA titer (g/L)	3.1 ± 0.7	9.3 ± 0.9
MA in aqueous phase (g/L)	3.1 ± 0.7	8.1 ± 1.0
MA in organic phase (g/L)	−	8.3 ± 0.8
Total MA (g)	3.2 ± 0.5	12.3 ± 1.3
PCA (g)	0.24 ± 0.05	0.57 ± 0.10
Yield (mg_MA_/g_glucose_)	30.4 ± 2.9	49.0 ± 4.4
Productivity (mg_MA_ /L/h)	16.5 ± 3.8	49.4 ± 4.6
Peak productivity (mg_MA_/L/h)	53.9 ± 6.1	100.0 ± 5.5
Cell dry weight (g/L)	12.9 ± 0.4	17.1 ± 0.5

**Table 2:** Overview of strains producing muconic acid *de novo* from carbohydrates.

Microbial Host	MA Titer (g/L)	Productivity (g/L/h)	Yield (mol_MA_/mol_substrate_)	pH	Reference
*Corynebacterium glutamicum*	88.2	2.00	0.30	7.0	[7]
	53.8	0.34	0.25	7.3	[24]
	4.5	0.06	0.22	−	[25]
*Pseudomonas putida*	33.7	0.18	0.46	7.0	[26]
	22.0	0.21	0.35	7.0	[27]
	13.0	0.26	0.27	7.0	[28]
*Pseudomonas occidentalis*	38.8	0.17	0.51	6.0	[11]
	7.2	0.07	−	4.0	[11]
*Escherichia coli*	64.5	0.54	−	7.0	[29]
	36.8	0.81	0.22	7.0	[30]
	4.45	0.12	0.26	7.0	[31]
*Saccharomyces cerevisiae*	22.5	0.19	0.10	6.0	[12]
	20.8	0.14	0.08	6.0	[13]
	5.1	0.03	0.07	5.0	[32]
	2.0	0.02	0.02	6.0	[33]
	1.2	0.01	0.04	6.3	[34]
	2.1	<0.01	0.02	5.0	[35]
	4.5	0.02	0.04	3.5	[17]
	4.3	0.03	−	4.0	[18]
	9.3	0.05	0.06	4.0	This study

**Table 3:** Plasmids used in this study.

Name	Description	Source
pCR2.1	Base plasmid for overexpression constructs	Thermo Fisher Scientific
pCR2.1-TEF1p_PDC5_ADH1t	Donor DNA template for *TEF1p_PDC5*	This study
pCR2.1-RPL18Bp_PDC5_ADH1t	Donor DNA template for *RPL18Bp_PDC5*	This study
pCR2.1-SAC6p_PDC5_ADH1t	Donor DNA template for *SAC6p_PDC5*	This study
pCR2.1-REV1p_PDC5_ADH1t	Donor DNA template for *REV1p_PDC5*	This study
pCR2.1-TEF1p_QDR3_ADH1t	Donor DNA template for *TEF1p_QDR3*	This study
pCR2.1-RPL18Bp_QDR3_ADH1t	Donor DNA template for *RPL18Bp_QDR3*	This study
pCR2.1-SAC6p_QDR3_ADH1t	Donor DNA template for *SAC6p_QDR3*	This study
pCR2.1-REV1p_QDR3_ADH1t	Donor DNA template for *REV1p_QDR3*	This study

**Table 4:** *S. cerevisiae* strains used in this study.

Name	Description	Source
TN22	MA platform strain	[17]
TN22 *TEF1p_PDC5*	*TEF1p_PDC5* integrated at IS12.2	This study
TN22 *RPL18Bp_PDC5*	*RPL18B1p_PDC5* integrated at IS12.2	This study
TN22 *SAC6p_PDC5*	*SAC6p_PDC5* integrated at IS12.2	This study
TN22 *REV1p_PDC5*	*REV1p_PDC5* integrated at IS12.2	This study
TN22 *TEF1p_QDR3*	*TEF1p_QDR3* integrated at IS4.2	This study
TN22 *RPL18Bp_QDR3*	*RPL18Bp_QDR3* integrated at IS4.2	This study
TN22 *SAC6p_QDR3*	*SAC6p_QDR3* integrated at IS4.2	This study
TN22 *REV1p_QDR3*	*REV1p_QDR3* integrated at IS4.2	This study
TN22 *RPL18Bp_PDC5; TEF1p_QDR3*	*RPL18B1p_PDC5* integrated at IS12.2;*TEF1p_QDR3* integrated at IS4.2	This study

## Data Availability

The original contributions presented in the study are included in the article/Supplementary Material, further inquiries can be directed to the corresponding author.

## Supplementary Materials

The following supporting information can be downloaded at: https://www.mdpi.com/article/10.3390/ijms251910245/s1.

## Abbreviations

ALE	adaptive laboratory evolution
ccMA	*cis*,*cis*-muconic acid
CDO	catechol 1,2 dioxygenase
CDW	cell dry weight
DHS	3-dehydroshikimate
HPLC	high-performance liquid chromatography
ISPR	*in situ* product recovery
MA	muconic acid
PCA	protocatechuic acid
PCAD	protocatechuic acid decarboxylase
Pdc^-^	pyruvate-decarboxylase negative
prFMN	prenylated flavine mononucleotide
RID	refractive index detector
YPD	yeast extract-peptone-dextrose
YPX	yeast extract-peptone-xylose
