# Supplementary material for: Metabolic Engineering and Process Intensification for Muconic Acid Production Using Saccharomyces cerevisiae
Source: Int J Mol Sci. 2024 Sep 24;25(19):10245. doi: 10.3390/ijms251910245 (PMC11476194; doi:10.3390/ijms251910245)
Supplement: Supplementary file 1 [file ijms-25-10245-s001.zip › ijms-3156218-supplementary.pdf]

Supporting information

Metabolic engineering and process intensification for muconic acid production using industrial yeast

Sinah Tönjes, Evelien Uitterhaegen, Ilse Palmans, Birthe Ibach, Karel De Winter*, Patrick Van Dijck, Wim Soetaert and Paul Vandecruys

Table S1. Plasmids used in this study.

Name	Sequence	Goal
H1 - XII-2	ACGGGCAATCAGAATCTGTAACAAGCGCCATTTTTTTTCTGTATCGGGCCCTCCTTACTGCTCTCCTTCATCAAGCTTGGTACCGAGC	Amplification of overexpression cassettes for integration in site IS12.2
H2 - XII-2	CCAAGTGGCAAAAGCGTTAGACGCAGTACAAGGACGCGTTAAGAAAAATTTCGAGAGAGTCGCCGATAGCTCACTATAGGGCGAATTGG	Amplification of overexpression cassettes for integration in site IS12.2
H1 - IS4.2	TATGTTTTCTTTCTAAAAAATCTTGAACATGGTCGCGACAGCTACTTTGACAATCATTGATAACATCAAGCTTGGTACCGAGC	Amplification of overexpression cassettes for integration in site IS4.2
H2 - IS4.2	TACGGCTTTCCCCCCTCTTTATTCGTACAGAAAACTTTACGCTCTGGCTTCTTCTGCTCATTTCTCACTATAGGGCGAATTGG	Amplification of overexpression cassettes for integration in site IS4.2
FW TEF1prom - overlap pCR2.1	CGCCAAGCTTGGTACCGAGCCCTTGCCAACAGGGAGTTCT	Amplification of *TEF1p*
RV TEF1prom - PDC5	CCTAAGGTTATTTCAGACATTTTGTAATTAAAACTTAGAT	Amplification of *TEF1p*
FW RPL18Bprom - overlap pCR2.1	CGCCAAGCTTGGTACCGAGCAAGAGGATGTCCAATATTTT	Amplification of *RPL18Bp*
RV RPL18Bprom - PDC5	CCTAAGGTTATTTCAGACATTTTGTTTTTTGTTTTCTTCT	Amplification of *RPL18Bp*
FW SAC6prom - overlap pCR2.1	CGCCAAGCTTGGTACCGAGCTTTGAGAATGACCTTCCTCG	Amplification of *SAC6p*
RV SAC6prom - PDC5	CCTAAGGTTATTTCAGACATTGTGTTTTGGTGTACTCCTT	Amplification of *SAC6p*
FW REV1prom - overlap pCR2.1	CGCCAAGCTTGGTACCGAGCGATACAACCGGATATTTTTC	Amplification of *REV1p*
RV REVprom - PDC5	CCTAAGGTTATTTCAGACATCGCTGGATATGCCTAGAAAT	Amplification of *REV1p*
FW PDC5	ATGTCTGAAATAACCTTAGG	Amplification of *PDC5* from genomic DNA
RV PDC5	TTATTGTTTAGCGTTAGTAG	Amplification of *PDC5* from genomic DNA
FW ADH1term - PDC5	CTACTAACGCTAAACAATAAGCGAATTTCTTATGATTTAT	Amplification of *ADH1t*
RV ADH1term - overlap pCR2.1	CTATAGGGCGAATTGGGCCCGAAATGGGGAGCGATTTGCA	Amplification of *ADH1t*
FW TEF1prom - overlap pCR2.1	CGCCAAGCTTGGTACCGAGCCCTTGCCAACAGGGAGTTCT	Amplification of *TEF1p*
RV TEF1prom - QDR3	TGTGAACCTTGGGCTTGCATTTTGTAATTAAAACTTAGAT	Amplification of *TEF1p*
FW RPL18Bprom - overlap pCR2.1	CGCCAAGCTTGGTACCGAGCAAGAGGATGTCCAATATTTT	Amplification of *RPL18Bp*

RV RPL18Bprom - QDR3	TGTGAACCTTGGGCTTGCATTTTGTTTTTTGTTTTCTTCT	Amplification of *RPL18Bp*
FW SAC6prom - overlap pCR2.1	CGCCAAGCTTGGTACCGAGCTTTGAGAATGACCTTCCTCG	Amplification of *SAC6p*
RV SAC6prom - QDR3	TGTGAACCTTGGGCTTGCATTGTGTTTTGGTGTACTCCTT	Amplification of *SAC6p*
FW REV1prom - overlap pCR2.1	CGCCAAGCTTGGTACCGAGCGATACAACCGGATATTTTTC	Amplification of *REV1p*
RV REVprom - QDR3	TGTGAACCTTGGGCTTGCATCGCTGGATATGCCTAGAAAT	Amplification of *REV1p*
FW QDR3	ATGCAAGCCCAAGGTTCACA	Amplification of *QDR3* from genomic DNA
RV QDR3	TTAATCAATTTTGTCGTACA	Amplification of *QDR3* from genomic DNA
FW ADH1term - QDR3	TGTACGACAAAATTGATTAAGCGAATTTCTTATGATTTAT	Amplification of *ADH1t*
RV ADH1term - overlap pCR2.1	CTATAGGGCGAATTGGGCCCGAAATGGGGAGCGATTTGCA	Amplification of *ADH1t*
